# Translatability of a Wearable Technology Intervention to Increase Adolescent Physical Activity: Mixed Methods Implementation Evaluation

**DOI:** 10.2196/13573

**Published:** 2020-08-07

**Authors:** Harriet Koorts, Jo Salmon, Anna Timperio, Kylie Ball, Susie Macfarlane, Samuel K Lai, Helen Brown, Stephanie E Chappel, Marina Lewis, Nicola D Ridgers

**Affiliations:** 1 School of Exercise and Nutrition Sciences Institute for Physical Activity and Nutrition Deakin University Burwood Australia; 2 Learning Futures Deakin University Geelong Australia; 3 School of Exercise and Nutrition Sciences Deakin University Geelong Australia

**Keywords:** wearable technology, social media, implementation science, adolescent, physical activity, awareness

## Abstract

**Background:**

Wearable technology interventions combined with digital behavior change resources provide opportunities to increase physical activity in adolescents. The implementation of such interventions in real-world settings is unknown. The Raising Awareness of Physical Activity (RAW-PA) study was a 12-week cluster randomized controlled trial targeting inactive adolescents attending schools in socioeconomically disadvantaged areas of Melbourne, Australia. The aim was to increase moderate- to vigorous-intensity physical activity using (1) a wrist-worn Fitbit Flex and app, (2) weekly challenges, (3) digital behavior change resources, and (4) email or text message alerts.

**Objective:**

This paper presents adolescents’ and teachers’ perceptions of RAW-PA in relation to program acceptability, feasibility and perceived impact, adolescent engagement and adherence, and the potential for future scale-up.

**Methods:**

A mixed methods evaluation of the RAW-PA study assessed acceptability, engagement, feasibility, adherence, and perceived impact. A total of 9 intervention schools and 144 intervention adolescents were recruited. Only adolescents and teachers (n=17) in the intervention group were included in the analysis. Adolescents completed web-based surveys at baseline and surveys and focus groups postintervention. Teachers participated in interviews postintervention. Facebook data tracked engagement with web-based resources. Descriptive statistics were reported by sex. Qualitative data were analyzed thematically.

**Results:**

Survey data were collected from 142 adolescents at baseline (mean age 13.7 years, SD 0.4 years; 51% males) and 132 adolescents postintervention. A total of 15 focus groups (n=124) and 9 interviews (n=17) were conducted. RAW-PA had good acceptability among adolescents and teachers. Adolescents perceived the intervention content as easy to understand (100/120, 83.3%) and the Fitbit easy to use (112/120; 93.3%). Half of the adolescents perceived the text messages to be useful (61/120; 50.8%), whereas 47.5% (57/120) liked the weekly challenges and 38.3% (46/120) liked the Facebook videos. Facebook engagement declined over time; only 18.6% (22/118) of adolescents self-reported wearing the Fitbit Flex daily postintervention. Adolescents perceived the Fitbit Flex to increase their physical activity motivation (85/120, 70.8%) and awareness (93/119, 78.2%). The web-based delivery facilitated implementation of the intervention, although school-level policies restricting phone use were perceived as potential inhibitors to program roll-out.

**Conclusions:**

RAW-PA showed good acceptability among adolescents attending schools in socioeconomically disadvantaged areas and their teachers. Low levels of teacher burden enhanced their perceptions concerning the feasibility of intervention delivery. Although adolescents perceived that RAW-PA had short-term positive effects on their motivation to be physically active, adolescent adherence and engagement were low. Future research exploring the feasibility of different strategies to engage adolescents with wearable technology interventions and ways of maximizing system-level embeddedness of interventions in practice would greatly advance the field.

## Introduction

Physical activity is an important component of a healthy lifestyle, yet physical inactivity is a global pandemic with far-reaching consequences for health and well-being both now and in the future [1]. The benefits of physical activity in childhood and adolescence include reduced cardio-metabolic risk factors, improved body composition, and higher fitness [2]. However, global estimates suggest that over 80% of adolescents do not engage in the recommended 60 min of moderate- to vigorous-intensity physical activity (MVPA) every day [3], and steep declines in physical activity levels during adolescence are common [4]. This is particularly evident for adolescents living in areas of socioeconomic disadvantage, who are less likely to meet physical activity guidelines and are at greater risk of declining activity levels [5]. There is clearly a need to identify strategies for maintaining - if not increasing - adolescent physical activity levels. However, compared with interventions targeting primary school children, few have been conducted, particularly with those living in socioeconomically disadvantaged areas [6].

One approach that may have the potential to promote physical activity levels in adolescents is the use of wearable activity trackers, which are electronic devices that are designed to be worn on the body that use sensors (eg, accelerometers) to track movement and/or biometric data [7]. These technologies enable constant self-monitoring of physical activity through the provision of data and feedback via a visual display and/or an accompanying app [8]. There is a dearth of data about wearable activity tracker ownership in adolescents, although one study found that approximately 25% of adolescents owned such devices, with more males than females reporting ownership [9]. Of note, school-based research has shown that wearable activity monitors have moderate acceptability among school-aged children in low-income communities [10]. Furthermore, there is some initial evidence that adolescents are generally positive about the use of wearable technology for tracking physical activity, and use device features to set goals and undertake challenges against friends [11]. From a school's perspective, wearable technology interventions have the potential to be highly implementable, given the low burden placed on teachers to deliver and potential minimal interference with existing school practices [10].

Wearable technology has recently been combined with social media as a platform to disseminate health-promoting messages and effect behavior change. Evidence from adults in a clinical setting has shown that wearable technology combined with a social media–based health education intervention increased daily light-intensity physical activity and MVPA [12]. Adolescents are known to be high users of social media [13], and those from low-income families report higher social media use than those from high-income homes [14]. The potential reach of such combined interventions among youth living in socioeconomically disadvantaged areas may comprise an opportunity to increase activity levels among these groups. Although it is known that adolescent males and females differ in their preferences for types of physical activities [15], less is known about how youth respond to technologies promoting physical activity, such as wearable activity trackers and digital behavior change resources, and if different social media strategies are more effective for engaging males or females. Physical activity apps, including web-based platforms, have promising reach and a low burden; however, there is limited evidence of their efficacy in adolescent populations [16].

To shift population-level physical activity, not only must interventions demonstrate effectiveness, but they must be sustainably implemented over time and under real-world conditions [17]. Physical activity interventions that are designed with real-world implementation and scale-up in mind are recommended [17]. However, most wearable device physical activity intervention studies, regardless of age group, are delivered in small samples (eg, <100 participants), and there is a dearth of studies in youth populations [18,19]. In a number of cases, particularly among clinical populations, participants are prescribed how to use the devices (eg, via a counseling study approach) [20,21], and implementation of wearable technology interventions in nonclinical settings and among nonclinical populations is, therefore, less well understood. “Furthermore, the majority of school-based studies investigating the impact and feasibility of wearable activity trackers on physical activity in youth have not been tested under real-world conditions and over longer periods (eg, >3 months), which may subsequently impede implementation and effectiveness [22].

This study aimed to assess the implementation of a wearable technology intervention to increase physical activity among adolescents: The Raising Awareness of Physical Activity (RAW-PA) study.

The specific aims of this study were to evaluate adolescent (individual level) and teacher (individual and school level) perceptions of intervention acceptability, feasibility and perceived impact, and adolescent engagement in and adherence to the intervention. Findings from this evaluation will provide important first evidence for the feasible real-world implementation of wearable technology interventions among inactive adolescents and provide recommendations to potentially enhance future implementation of such interventions if delivered on a larger scale.

## Methods

### Overview of Raising Awareness of Physical Activity

A detailed description of the program and study protocol has been published elsewhere (ANZCTR: ACTRN12616000899448) [22]. In brief, RAW-PA was a 12-week cluster randomized controlled trial conducted in 2016-2018, which targeted adolescents (year 8, ie, second year of secondary school) attending schools in socioeconomically disadvantaged areas of Melbourne, Australia. After the 12-week intervention period, the intervention ceased as intended. RAW-PA combined both wearable technology and digital behavior change resources accessible via social media. These types of combined interventions can be known as *digital behavior change interventions*; however, given that this term can include a broad range of intervention types, for the purposes of this paper, we refer to RAW-PA as a *wearable technology intervention*. RAW-PA was co-designed (eg, style and frequency of delivery) with the target users (adolescents) [22], and it incorporated low-cost strategies to facilitate real-world implementation and the potential for wider scale-up [22]. Based on the social cognitive theory [23] and behavioral choice theory [24], the intervention promoted awareness of physical activity via a wearable physical activity tracker and accompanying app, and focused on increasing activity levels using digital behavior change resources.

RAW-PA aimed to increase adolescent MVPA by targeting the accumulation of activity across the day, which included out-of-school hours and weekends. Core components of the intervention included: (1) a wrist-worn Fitbit Flex and accompanying Fitbit app; (2) interactive weekly individual and/or team *missions* or *challenges* (delivered via email and/or text message approximately 2-3 times/week); (3) digital behavior change resources (eg, motivational videos and social forums accessible via a private Facebook group); and (4) email and/or text message alerts to new content, missions, or challenges (approximately 2-3 times/week). One of the weekly challenges (*Mark it Up!*) focused on increasing awareness of activity opportunities in schools where students and teachers identified and shared strategies for increasing activity at school and competed in a step challenge against each other. To facilitate this challenge, 2 teachers from each intervention school were provided with a Fitbit Flex. The remaining challenges targeted behavior change outside of school, and content was delivered during out-of-school hours [22]. The trial adhered to the consolidated standards of reporting trials guidelines, and ethical approval was obtained from the Deakin University human research ethics committee (2016-179) and the Victorian Department of Education and Training. Participating schools provided written informed consent, and parents provided signed consent, which included student assent.

### Evaluation Design

This study used a mixed methods evaluation design based on the UK Medical Research Council recommendations [25]. A total of 5 evaluation indicators were identified based on recommended outcomes for process evaluation and implementation-related research [25,26]: (1) *acceptability* (eg, adolescent enjoyment, ease of understanding and Fitbit use, and teacher-perceived barriers to uptake), (2) *engagement* (eg, frequency of adolescent interaction with Facebook group/posts), (3) *feasibility* (eg, adolescent barriers to Fitbit wear and teacher-perceived suitability of delivery in the school setting), (4) *adherence* (eg, adolescent self-reported completion of weekly challenges), and (5) *perceived impact* (eg, perceived changes in motivation, awareness, and encouragement for physical activity).

### Participants and Recruitment

**Figure 1:**
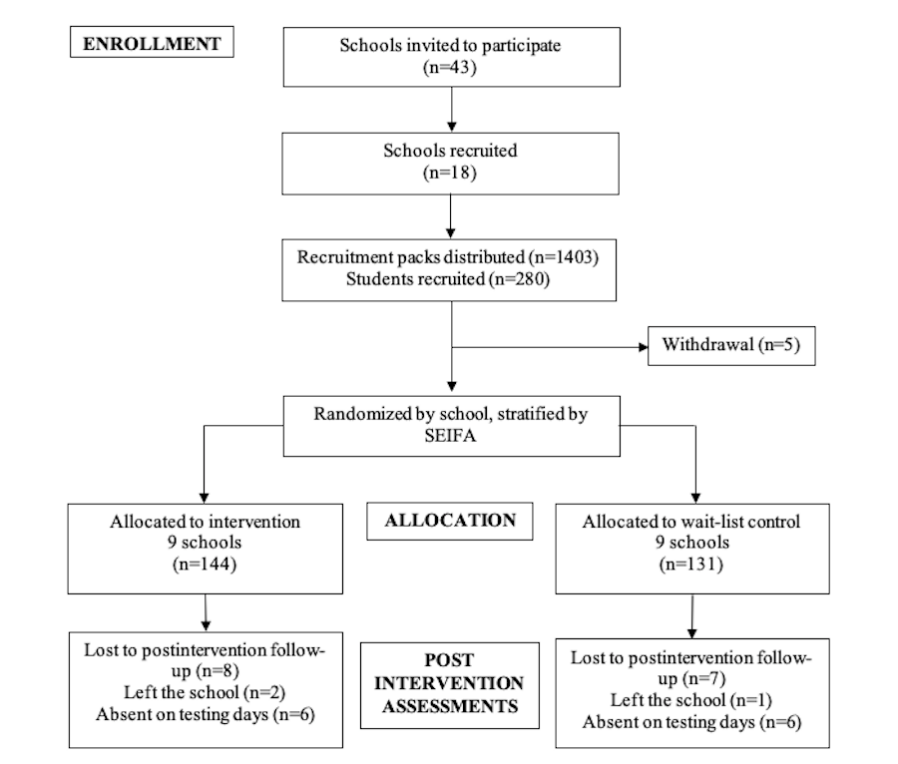
Flow of participants. SEIFA: socioeconomic index for areas.

Adolescents were recruited through the school setting for ease of recruitment. In total, 18 schools (intervention: n=9 and wait-list control: n=9) and 280 students were recruited. A total of 5 participants withdrew before baseline data collection; therefore, 275 students (intervention=144 and wait-list control=131) took part in the study (Figure 1). Schools were eligible to participate in the study if they were located in areas that had a score of ≤5 (lowest 50%) on the socioeconomic indexes for areas (SEIFA [27]) within 60 km of Deakin University’s Burwood Campus. Eligible schools were randomly selected to receive an invitation to participate in the study. The year eight coordinators were invited to be the school liaison and help with data collection. Year coordinators, as opposed to classroom teachers, were recruited as they typically had contact with all students in the year group. Schools that provided written informed consent from their school principal to participate in the study were matched based on SEIFA score and size and randomly assigned to either the intervention or wait-list control group by a computer-based random number generator [22]. All participating schools were located in urban areas. Recruitment and baseline data collection were conducted before school randomization.

For eligibility, adolescents were required to (1) be at least 13 years old (minimum age required to have a Fitbit and a Facebook account); (2) not engage in regular organized physical activity sports outside of school; (3) not meet national physical activity guidelines of at least 60 min of MVPA daily [28]; (4) not be a current or past owner of an activity tracker; (5) have, or be willing to create, a Facebook account; and (6) have access to the internet outside of school (eg a mobile device with data or Wi-Fi at home). A self-report checklist containing these eligibility criteria was included with each parental consent form, which was completed by parents and students. Each student who returned a completed parental consent form (which included student assent) and met the eligibility criteria was recruited into the study. Participants in the wait-list control group were provided access to the intervention materials on completion of the 6-month follow-up assessments, but no implementation/process data were collected.

### Procedure

All adolescents participating in the study were invited to complete a web-based survey at baseline and postintervention (12 weeks after baseline). Adolescents completed the web-based survey using iPads within school hours. Hard copies of the survey were provided to schools for those adolescents absent on the day of data collection at baseline (n=13) and postintervention (n=29) and were completed in the presence of teachers in the intervention schools. Due to modem failure, 23 adolescents completed hard copies of the survey at postintervention. Adolescents attending schools randomized to the intervention group were additionally invited to participate in a focus group (ranging between 4 and 13 students per group) at postintervention to explore their perspectives on RAW-PA [22]. Teachers from intervention schools were also invited to participate in an interview postintervention to provide insights about the approach from an organizational perspective. All interviews and focus groups were conducted on-site during school hours. Discussions followed a semistructured format and were audio recorded. The 15 focus groups (mean duration 26 min, SD 5 min) and 9 interviews (mean duration 21, min SD 7 min) were then transcribed verbatim for further analysis.

### Measures

Table 1 summarizes the relevant data for the 5 implementation evaluation indicators included in this study, collected postintervention. Baseline adolescent surveys captured participant reach and sociodemographic variables. A complete and detailed list of measures has been previously published [22].

*Quantitative survey data* were used to assess adolescent-perceived acceptability (11 questions), feasibility (3 questions), adherence (3 questions), and perceived impact (6 questions) of RAW-PA. Items were rated on a 5-point Likert scale from strongly disagree (1) to strongly agree (5), with 1 question requiring reverse coding (*Please indicate the extent to which you agree with the following statements: Wearing the Fitbit was uncomfortable and I felt embarrassed wearing the Fitbit*). Qualitative free-text survey responses contributed to assessing acceptability (3 questions: what adolescents liked most and least, and suggested improvements) and feasibility (1 question: reasons for suggesting a different intervention duration; Table 1) and adherence (1 question: reasons for Fitbit nonwear). These items were included in the intervention group surveys only.

*Qualitative focus group and interview data* provided information about adolescent- and teacher-perceived program acceptability, feasibility, adherence, and impact. Exemplar questions are shown in Table 1.

*Web-based Facebook data* of *views* and *likes* were recorded weekly during the 12-week intervention to assess the frequency of adolescent engagement with the Facebook group. As the research team posted comments within the Facebook group in addition to participants, only *views* (ie, posts clicked on and viewed) and *likes* (ie, participant *liked* a post) of the web-based program material were used to assess engagement.

**Table 1:** Implementation evaluation indicators and assessment criteria postintervention.

Evaluation indicator and assessment criteria		Data source		Exemplar question/reporting criteria	
**Acceptability**					
	Adolescent-perceived intervention enjoyment, ease of understanding/use, and comfort wearing a Fitbit		Web-based survey (14 questions)		“The information was easy to understand.” “Wearing the Fitbit was uncomfortable.”
	Adolescent levels of intervention enjoyment and challenges faced		Focus group (6 questions)		“Did you experience any issues when using/accessing features of the program?”
	Teacher-perceived barriers and facilitators to intervention uptake and perceptions of acceptability		Interview (5 questions)		“How acceptable would such a program be to schools?”
**Engagement**					
	Frequency of *views* and *likes* of intervention strategy Facebook posts^a^		Facebook data		Total number of *views* and changes over time (ie, the post was clicked on and viewed) and *likes* of each Facebook postadolescents received
**Feasibility**					
	Adolescent-perceived appropriateness of the intervention duration		Web-based survey (4 questions)		“The length of the RAW-PA^b^ program was just right.”
	Barriers and facilitators to accessing the program		Focus group (3 questions)		“Did anything help you to use/access any feature(s) of the program?”
	Teacher-perceived appropriateness of intervention delivery in the school setting		Interview (2 questions)		“What considerations would schools make before participating in such a school-based challenge?”
**Adherence**					
	Adolescent self-reported adherence to wearing a Fitbit and completion of weekly challenges		Web-based survey (4 questions)		“On how many days did you wear the Fitbit in the last week?”
	Barriers and facilitators to wearing the Fitbit and adhering to the program		Focus group (1 question)		“Did anything stop you using/accessing any feature(s) of the program?”
**Perceived impact**					
	Adolescent-perceived impact of intervention on motivation, awareness, and encouragement for physical activity		Web-based survey (6 questions)		“The Fitbit motivated me to be more active.” “The Fitbit made me think about how much activity I do.”
	Adolescent-perceived impact of intervention and change in awareness regarding physical activity		Focus group (2 questions)		“Did the program change your awareness of your activity levels?”
	Teacher-perceived impact of the intervention on students and school and impact on teacher awareness of own physical activity		Interviews (3 questions)		“Did the program change your awareness of your own physical activity levels?”

^a^Web-based Facebook engagement was captured weekly during the 12-week intervention period.

^b^RAW-PA: Raising Awareness of Physical Activity Study.

### Analyses

Descriptive statistics from adolescent survey data were calculated for 4 of the 5 evaluation indicators by sex (acceptability, adherence, feasibility, and perceived impact). Survey data were combined into 3 groups, classified as *agree* (sum of responses *strongly agree* and *agree*), *neither* (sum of responses *neither agree* nor *disagree*) and *disagree* (sum of responses *strongly disagree* and *disagree*). This approach is appropriate for analyzing ordinal data and provides insights into the adolescents’ perspectives about the intervention [29]. Continuous sample characteristics are presented as means and SDs, and categorical data are presented as counts and percentages. The total *potential* number of weekly views/likes possible on Facebook was calculated based on the number of participants registered for the Facebook group multiplied by the number of posts provided to participants each week. This score was based on the assumption that every registered participant had the opportunity to view/like each post on at least one occasion per week. Restrictions on the information available for download via Facebook meant that Facebook data could not be stratified by sex. Sex differences in adolescent perceptions of intervention acceptability, adherence, feasibility, and perceived impact were calculated using a Mann-Whitney test. All analyses were conducted using Stata SE 15 (StataCorp LP).

Qualitative free-text survey responses were coded thematically. Transcribed qualitative data were imported into NVivo 12 (QRS International) for coding and thematic analysis. Thematic analysis requires initial data familiarization, coding, and tabulation of raw themes, which are then grouped based on patterns of emergence and overlapping relevance [30]. Coding and theme development were first deductive (theory driven), guided by the study aims, process evaluation framework [25], and project team’s previous research and conceptualization [11], followed by an inductive approach (data driven) directed by the content of the data [31]. Specifically, the process evaluation provided a metathematic structure to guide initial coding, and subthemes relating to the adolescents’ and teachers’ perceptions and experiences were then identified. Consistent with the recommended approaches [30], 2 researchers independent of the project team (ML and SC) were engaged to analyze the qualitative data. Selections of raw data were independently coded by the lead author (HK) by means of cooperative triangulation. Presented themes were critically questioned and interpretations of these data were challenged. Instances of divergence were discussed until consensus was reached. Illustrative quotes were extracted from the coded data to reflect and support the themes identified from these data.

## Results

### Overview

In the 9 intervention schools, 144 and 136 adolescents (Figure 1) were invited to complete the surveys at baseline and postintervention, respectively. Of the 8 students not invited at postintervention, 2 had left the school and 6 were absent. A total of 142 (99%) adolescents completed baseline surveys (mean age 13.7 years, SD 0.4 years; 51% males), and 132 (98%) adolescents completed surveys postintervention (mean age 14.0 years, SD 0.4 years; 52% males). Approximately 85% (n=122) of adolescents (52% males) registered for the Facebook group. The 15 focus groups conducted comprised 124 (86%) adolescents (51% males), and the 9 interviews involved 17 (81%) teachers (47% males).

### Acceptability

Table 2 presents the percentage of adolescents as well as the proportion of males and females who stated *agree* or *strongly agree* to questions relating to acceptability, feasibility, adherence, and perceived impact. The majority of adolescents reported that the program was easy to understand, enjoyable, and they would recommend it to their friends. Half of all adolescents thought the text messages were useful, and less than half liked the weekly challenges and Facebook videos. Males were significantly more likely than females to agree that they liked the Facebook pages and videos. With regard to the acceptability of the Fitbit Flex, almost all adolescents perceived that the Fitbit Flex was easy to use, and the majority agreed that they got used to wearing it and they did not feel embarrassed.

**Table 2:** Descriptive statistics of evaluation indicators postintervention.

Implementation evaluation indicators^a^			Overall n (%)		Male n (%)		Female n (%)		*P* value	
**Acceptability**										
	**Fitbit**									
		The Fitbit was easy to use (N^b^=120)		112 (93.3)		57 (91.9)		55 (94.8)		.54
		I got used to wearing the Fitbit (N^b^=119)		85 (71.4)		42 (68.9)		43 (74.1)		.49
		The Fitbit was comfortable to wear (N^b^=120)		70 (58.3)		37 (59.7)		33 (56.9)		.94
		I was not embarrassed wearing the Fitbit (N^b^=120)		94 (78.3)		48 (77.4)		46 (79.3)		.69
	**RAW-PA^c^ program**									
		The text messages were useful (N^b^=120)		61 (50.8)		34 (54.8)		27 (46.6)		.50
		I liked the Facebook page *(N*^*b*^*=120)*		*71 (59.2)*		*44 (71.0)*		*27 (46.6)*		*.03*
		Information was easy to understand (N^b^=120)		100 (83.3)		52 (83.9)		48 (82.8)		.94
		I liked the weekly challenges/missions (N^b^=120)		57 (47.5)		33 (53.2)		24 (41.4)		.22
		I liked the videos *(N*^*b*^*=120)*		*46 (38.3)*		*29 (46.8)*		*17 (29.3)*		*.03*
		I enjoyed the program (N^b^=120)		89 (74.2)		48 (77.4)		41 (70.7)		.32
		I would recommend the program to friends (N^b^=120)		85 (70.8)		48 (77.4)		37 (63.8)		.07
**Feasibility**										
	The program length was appropriate (N^b^=118)		74 (62.7)		36 (60.0)		38 (65.5)		.65	
**Adherence**										
	I completed the weekly challenges/missions (N^b^=119)		43 (36.1)		26 (42.6)		17 (29.3)		.13	
**Perceived impact**										
	**Fitbit**									
		Motivated me to be more active (N=120)		85 (70.8)		45 (72.6)		40 (69.0)		.68
		Made me think about how much activity I do (N^b^=119)		93 (78.2)		48 (77.4)		45 (79.0)		.80
	**RAW-PA program**									
		Challenges motivated me to be more active (N^b^=119)		41 (34.5)		24 (38.7)		17 (29.8)		.66
		Encouraged increased activity on own (N^b^=120)		74 (61.7)		40 (64.5)		34 (58.6)		.42
		Encouraged increased activity with family (*N*^*b*^*=119*)		*48 (40.3)*		*31 (50.0)*		*17 (29.8)*		*.05*
		Encouraged increased activity with friends (N^b^=120)		65 (54.2)		38 (61.3)		27 (46.6)		.16

^a^Data (%) reported is a combined score relating to those who stated *agree* and *strongly agree*. *P* value for sex differences significant at less than or equal to .05 (italics); calculated by Mann-Whitney tests.

^b^The N values differ due to questions not being completed in the surveys.

^c^RAW-PA: Raising Awareness of Physical Activity.

Responses to open-ended survey questions revealed that the aspects of the program participants liked most were receiving a free Fitbit (n=40) and the increased motivation to be active (n=28). The least preferred aspects included the Fitbit Flex design (eg, discomfort wearing and frequent need to charge the device, n=21) and the weekly challenges being too hard or demotivating (n=10). The frequency and volume of program notifications were also perceived negatively by a small number of participants (n=7), including receipt of text messages during class time.

During focus groups, a number of subthemes emerged as influencing perceptions of acceptability. Adolescents referred positively to specific features of the Fitbit Flex, such as the monitoring of sleep and physical activity:

I like how it records your steps because then at lunch you look at it and you can slowly start to improve your steps and then yep, it works out, it evolves and yeah, it’s good. I really liked that.

adolescent, school A

Adolescents also described the visual feedback on physical activity performance from the Fitbit Flex and Fitbit app as motivating:

Sometimes at the end of the day I want to get two dots and then next day I tried to like, get five dots.

adolescent, school J

And it [Fitbit app] also said...it also said like, which day you are the most active and which day is the worst so you can just like try to be more active like, every day.

adolescent, school A

Adolescents also referred positively to the goal-setting component of RAW-PA via the Fitbit Flex and Facebook posts. The focus groups highlighted that the social aspects of the intervention, such as challenging and competing with peers, influenced their goal setting:

It shows like, how many steps I’ve taken in a day and it also encouraged me since like, there’s challenges where I can do it with my friends and try to beat them.

adolescent, school M

I like [RAW-PA] challenges to be honest. When I first got it [the challenge] I tried to be active.

adolescent, school J

Less favorable aspects of the program were those associated with the Fitbit Flex and app. This included technical difficulties connecting to the app and inaccuracies of device monitoring and feedback:

When you shake your hand a lot because—and I was playing drums and then it was really hard because every second it would vibrate. It was annoying so I had to take it off and playing the drums counted as a step.

adolescent, school A

A small proportion of adolescents referred to a lack of awareness of the Facebook notifications due to the amount of competing web-based information. This included negative perceptions about the number of notifications sent:

Well, I didn’t like how many notifications it sent, because if I want to check my - your email for something, all the notifications would come up instead.

adolescent, school O

Interviews with teachers revealed that RAW-PA was perceived as highly acceptable at the school level. Perceived enjoyment of the program by students was central to teachers' ongoing support, as well as the small amount of teacher time and investment required to support program delivery. This ease of use increased the acceptability and support for RAW-PA in schools:

But once it was set up with the small group that did end up participating, then it was minimal amount of my time, which was fantastic...So that made it easy for me to have it running in the school, and easy to support it.

teacher, school K

One teacher referred to the personal benefits of increased awareness of their physical activity as a result of the Fitbit challenges:

[For] *me personally, it probably got me off my bum a little bit. I enjoyed the challenge with the kids, and I still wear it daily, and I’ve been monitoring, that I am getting my minimum 10,000. I’m getting beyond that each day, but I’m more conscious of that. So from a personal level, it was good for me.* [teacher, school N]

The teachers perceived that role modeling physical activity was positively associated with student engagement:

It motivated the students a little bit more. I did see that there was a little bit more engagement with students with the teachers involved.

teacher, school I

It was really good to actually get the teachers involved into the program, because the students are also affected by what we do, and if we sort of role model it to them they’re more likely to get engaged and involved in the activity as well.

teacher, school K

Teachers acknowledged the advantages of their reduced involvement in the implementation of RAW-PA in minimizing any potential increase in their existing workloads. Limited teacher involvement was also perceived to increase adolescent autonomy and responsibility:

They had to actually commit to something, which they’d never been asked to before...it gave them a bit of responsibility that they’ve not had before.

teacher, school N

Nonetheless, teachers equally acknowledged that their lack of involvement in the Facebook group meant they had no awareness of what the program was promoting via Facebook and what interactions were taking place. The 4 teachers described that the lack of feedback impacted both their own participation and their knowledge of what was happening among the students:

So it [the program] didn’t maybe like, get us, like, had a major impact on me...But, yeah, if we had like, kids talking to us, or we at least like, knowing who won, and yeah, but no one really talked to us, and we’re like OK.

teacher, school M

Although RAW-PA was not designed as a school-based physical activity intervention, teachers consistently described wanting greater information regarding the content of the program material and awareness of their students’ involvement and performance:

I wish that we had a bit more like information of how the kids were going...like the steps, about our challenge, so like, if we could see that, let’s say, I don’t know, like a newsletter...

teacher, school M

So maybe some more teacher involvement just to, sort of checking in and getting feedback, and being a little bit more active in the kids who are in the programme is probably needed I’d say.

teacher, school K

If implemented at scale, challenges highlighted by teachers included the need for increased responsibility at the school level, including how the program would be funded. Sustainable implementation was suggested to require stronger links with the community to achieve broader program acceptability:

Schools are very big on community involvement, so whether it’s linked to the school, I don’t know if you can use the school Facebook page as well and give updates through that.

teacher, school I

### Engagement

**Figure 2:**
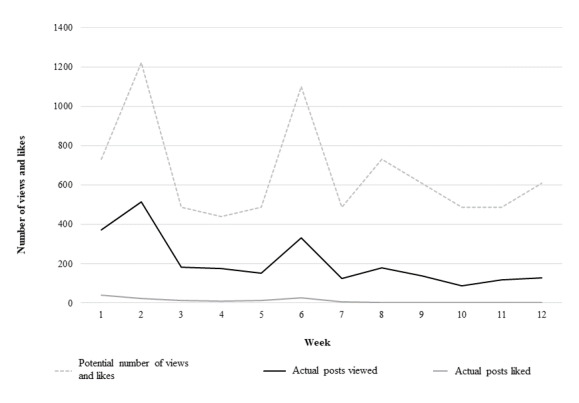
Number of Facebook posts viewed and liked by adolescents. The potential number of views and likes was based on the total number of participants registered for the Facebook group (n=122) multiplied by the number of posts provided on Facebook each week during the intervention. Actual posts liked and viewed tracked by Facebook.

Engagement in RAW-PA was based on the frequency that the Facebook posts were *liked* and/or *viewed* by participants registered for the Facebook group. In general, more posts were viewed than liked. Engagement in the Facebook group declined over time (Figure 2). In week 1, there was the potential for at least 732 views and/or likes of 6 different Facebook posts that were made (Figure 2); however, adolescents viewed and liked the posts only 370 and 40 times in week 1, respectively.

### Feasibility

Irrespective of sex, the majority of adolescents agreed that the 12-week intervention duration was appropriate (Table 2). A total of 23 (20%) adolescents did not think the 12-week duration was an appropriate length, and of these, the majority preferred a 6-week program (Table 3). A preference for intervention duration did not differ between males and females. The most frequent reason for preferring a shorter duration was to retain participant motivation in the program and subsequent Fitbit use (n=9).

**Table 3:** Preferred intervention duration for adolescents who did not agree with the 12-week duration (N=23).

Preferred intervention duration	Overall (n=23), n (%)	Males (n=9), n (%)	Females (n=14), n (%)
4 weeks	2 (8)	0 (0)	2 (14)
6 weeks	7 (30)	5 (55)	2 (14)
8 weeks	5 (21)	3 (33)	2 (14)
10 weeks	5 (21)	1 (11)	4 (28)
16 weeks	2 (8)	0 (0)	2 (14)
20 weeks	2 (8)	0 (0)	2 (14)

During the focus groups, however, adolescents most frequently stated that the 12-week duration was too long and that they would prefer a program duration of 5 to 10 weeks. This was often justified based on the declining motivation to participate over time:

I feel like the program was a bit long. I feel like a lot of people started to get less motivated around week ten than week eight.

adolescent, school N

A number of subthemes emerged as influencing perceptions of intervention feasibility. These were characteristics of the intervention design and factors within the home and school environment. In relation to intervention design, program reminders via Facebook and the Fitbit app meant RAW-PA was freely accessible by adolescents in their leisure-time outside of school hours. One adolescent referred to family involvement as a key facilitator of participation:

My brother would take me training...he said oh he’d really like to, so he started taking me out nearly every single day playing basketball, just doing drills on the oval and stuff.

adolescent, school J

The potential feasibility of RAW-PA was hindered by technical issues such as Fitbit Flex syncing and charging, challenges accessing the app, and the requirement for internet access. Some adolescents reported having limited access to Wi-Fi and limited mobile phone data plans, which made accessing intervention content challenging. Adolescents described these as potentially having a negative impact on their sustained engagement and program impact:

Oh, sometimes when I had like, five dots on my Fitbit, at around nine o’clock at night it would just reset back to zero steps, and that was quite annoying.

adolescent, school O

My Fitbit wouldn’t connect to the app so when I just like went on the app it wouldn’t tell me how many steps I took and stuff.

adolescent, school I

I have a limited amount of WiFi.

adolescent, school F

Despite technical difficulties experienced with the technology, teachers described this *hands off* intervention design as a major facilitator of implementation and program feasibility:

So if we’ve got the freedom to manipulate the program around what we’re doing, it’s a whole lot easier than us having to stop everything to fit the program in. That would have been almost impossible, and I think it would have fallen over.

teacher, school N

Nonetheless, there was mixed support for the participants’ use of phones during the school day. During the intervention period, teachers allowed students to access social media (ie, to receive RAW-PA notifications); however, most teachers indicated that the use of phones was not permitted during class time. Although phone use during recess and lunchtime was mostly unrestricted, one teacher described their school attempting to extend restrictions on screen use to include recess:

Well at recess we’re trying to limit the screen time on iPads, and so we’re not encouraging them to use it [during recess] either.

teacher, school M

One teacher described the potential negative implications for future uptake and implementation of the program as a result:

I think that if you came to a school and said, “We’d be requiring the kids to be checking on their Facebook during the school day,” you’d find the school a lot less enthusiastic about it.

teacher, school J

To increase potential feasible implementation, several teachers suggested that RAW-PA could be introduced during a dedicated physical education lesson or integrated as part of the existing health and physical education (HPE) curriculum.

### Adherence

Adherence to RAW-PA was evaluated based on self-reported adherence to wearing the Fitbit Flex and the completion of weekly challenges, including self-reported barriers and facilitators. In the surveys, 18.6% (22/118) of adolescents reported wearing the Fitbit Flex daily in the last week of the program and 35.5% (42/118) reported not wearing it at all. A total 22.4% (13/58) of females and 15.0% (9/60) of males wore the Fitbit on all 7 days. There were no significant sex differences in Fitbit wear. The main reasons for failing to wear the Fitbit included forgetting to wear it (50/97, 51.5%), forgetting to charge it (39/97, 40.2%), and having a flat battery (36/97, 37.1%). The most common *other* reason for nonwear was losing the charger (n=6). Approximately one-third of adolescents reported completing the challenges during the 12 weeks (Table 2).

Consistent with survey data, the most commonly reported barrier to Fitbit wear in the focus groups was the loss of the device and/or charger. For example, an adolescent referred to a subsequent lack of interest in continuing to use the Fitbit after a period of not wearing it:

I wore it [the Fitbit] for like, the first nine weeks and then I lost it. Then I found it again then I wasn’t interested in wearing it again so I just didn’t wear it.

adolescent, school O

One of the main reasons for declining interest in adhering to the program over time was adolescents’ short-term motivation to achieve daily step goals:

I started off with 10,000 and then it was like, every day if I reach my goal, I’ll increase it by 1,000...But that was only like, during the first week or two and then eventually like, I just don’t care for it anymore. It’s just sort of, the interesting bit’s worn off.

adolescent, school N

### Perceived Impact

Surveys showed that the Fitbit Flex was perceived to have had a greater impact on the adolescents’ physical activity than other RAW-PA components (Table 2). Consistent for males and females, the majority of adolescents agreed that wearing the Fitbit Flex motivated them to be more active and increased their awareness of their own physical activity levels. The program encouraged the majority of adolescents to be more active on their own; however, only around one-third agreed that the weekly challenges motivated them to be more active. Males were significantly more likely than females to report that the program encouraged them to be more active with their families. During the focus groups, adolescents spoke positively regarding the perceived impact of RAW-PA and understood the aims of the intervention. Increased motivation to be active due to competing with peers was often described as having an impact on their physical activity:

I went shopping with my family, which I never do. We were going furniture shopping. I was like nope, got to get my steps up...I was walking around [name of shop] over and over and over, just to get the steps up, because I wanted to win.

adolescent, school N

However, for some adolescents, the competition aspect was in fact demotivating and had a negative impact:

At the start, I was like, okay I’m going to beat everyone...I wake up the next morning...and everyone’s already got like a million steps...how am I meant to overtake everyone? So I was like, well, there’s no hope.

adolescent, school N

When asked if they thought the program was successful, the majority of adolescents indicated that they did. Overall, adolescents reported a greater awareness of their physical activity levels as a result of RAW-PA:

I kind of noticed how much like, I actually moved around during the school day, because I’m in a lot of classes in lunch time and recess that I actually have to move around to cross the school.

adolescent, school O

Some adolescents reported that RAW-PA made them want to be more active, leading to potential increases in their physical activity levels:

It like, made you want to do more, like physical things.

adolescent, school F

I don’t really like to exercise, because I don’t think I’m very good at it. But since I got the Fitbit I’m actually bothering to exercise now...

adolescent, school O

However, there was some evidence that any changes in behavior were unlikely to be sustained:

The start when we got the Fitbit, the app round the start I had, I was very inactive. I slowly started getting more steps but then I started falling back down.

adolescent, school J

I don’t think it affected me that much. I mean, it - it increased it a little bit, but most of the time it just became normal and I just continued whatever my normal week would be.

adolescent, school O

Consistent with adolescents’ feedback, teachers agreed that the intervention was positive; they perceived it had raised adolescents’ awareness of physical activity and was therefore likely to be of benefit. However, the sustainability of any changes leading to long-term impact was questioned:

Yeah, two girls who normally weren’t very active had decided that they would start to do a gym program...It was a short term thing, but they did actually start to work together and try and push each other a little bit. So for those two girls, that’s extraordinary because they normally don’t do much activity.

teacher, school N

At the individual teacher level, 2 teachers reported increased awareness of their own physical activity and a subsequent change in their physical activity behavior as a result of participating:

The teacher challenge bit, you know, had an impact on me. Like, I’m still wearing it, and I’m still using it, and it’s not something I’ve done before.

teacher, school J

Well the impacts on myself and I could say my colleague as well were just that we were really aware of our activity to make sure that we were quite active.

teacher, school A

## Discussion

### Principal Findings

The RAW-PA program had good acceptability among inactive adolescents and was highly acceptable at the school level. Based on the qualitative and quantitative data, text messaging and weekly Facebook challenge components of the program were less popular, although the social aspects of peer challenges and competition were popular with some but not others. Technical difficulties with the Fitbit Flex and app hindered the adolescents’ experiences of the program, and adherence and engagement were generally low as a result of device loss and declining use of Facebook over time. Adolescents perceived that the Fitbit Flex increased their physical activity awareness and likelihood of being more active on their own in the short term, although competition with peers was associated with both increased and decreased motivation to be active. The web-based program delivery was perceived by teachers as a key facilitator of implementation and program feasibility in schools, although teachers wanted greater access to and awareness of the program content and student involvement. Teachers considered the program highly acceptable and feasible for the school setting; however, they highlighted that any future implementation may be significantly limited by school policies restricting mobile phone use within schools.

### Comparison With Prior Work

The findings from this study support previous research that shows that wearable technology interventions are both acceptable among adolescents [11] and feasible for implementation within the school setting [10]. Overall, there were few sex differences in adolescent males’ and females’ experiences and perceptions of RAW-PA, apart from males being significantly more likely than females to like the Facebook page and weekly videos. Co-designing digital health interventions with those affected by the issues of interest is associated with increased engagement in both research and real-world settings [32]. Despite adolescents having input into the RAW-PA program design before study implementation [22], including input into the format and content of the digital resources and program length, engagement with the RAW-PA Facebook group declined rapidly over time. Although adolescents reported a perceived short-term increase in their motivation for and awareness of physical activity resulting from the device, in line with previous research [33], adolescents’ interest in the Fitbit Flex and motivation to achieve daily step goals was also often short-lived and use was reported to be low at the end of the program. This is consistent with a study in the United Kingdom involving 100 adolescents (13- to 14-years old), which showed positive increases in physical activity motivation in response to wearing a Fitbit Charge for 8 weeks, although the effects were not maintained [33]. Although the majority of adolescents reported that the 12-week program was appropriate, these results suggest that shorter programs (eg, 6-8 weeks) may be needed to sustain engagement and interest, particularly for males.

It was also shown that approximately 60% of adolescents found that the Fitbit was comfortable to wear. Design and esthetics are important considerations for wearable activity trackers [34,35], as these can promote engagement and use of devices [18]. Although this device was trialed with adolescents before use [11], and contrasting findings were found in relation to comfort, this was mentioned less frequently than other potential issues such as knowing how to use the device. It is possible that the greater study length (12 weeks vs 6 weeks) may have impacted perceptions of comfort. Overall, this reinforces that comfort is an important consideration for wearable activity tracker interventions.

Overall, social aspects related to peer competition in RAW-PA were viewed positively by adolescents. Nonetheless, in focus groups, adolescents described the competitive goal-setting aspect of RAW-PA as having both a positive and negative effect on their motivation to be active. Peer social influences and support for physical activity are linked to adolescent physical activity [36,37], which may explain the positive experiences among some participants. However, the strength of this relationship via wearable technology and digital behavior change/social media interventions in youth is less clear. In adults, tracking of goals via the Fitbit Flex has been linked to increased motivation for changing physical activity behaviors [38], and web-based social networks have also been effective at increasing physical activity due to the promotion of social comparison (eg, competitive relationships) and support to motivate behavior change [39,40].

Although web-based networks can improve physical activity through social support for healthy behaviors [41], low levels of participant engagement and a lack of overall program adherence in this study make it difficult to draw conclusions regarding the role of peer social support on adolescent physical activity in this context. Nonetheless, the *forced* competitive elements of wearable technology interventions (eg, step count challenges via the Fitbit app) have been linked to a loss of autonomy in adolescents and reduced self-determined motivation to be active [33]. A study exploring the impact of an 8-week Fitbit intervention on 13- to 14-year old adolescents’ motivation to be physically active showed that adolescents’ autonomous motivation to be active was significantly reduced postintervention [33]. The competition resulting from ongoing self-monitoring via the Fitbit and app in RAW-PA may have negatively impacted some adolescents’ autonomy to be active and thus explains the mixed experiences relating to the competitive goal-setting aspects.

From a school-level perspective, however, low levels of burden experienced by teachers in RAW-PA increased program acceptability and feasibility. This was expected given that common barriers to physical activity intervention implementation include, for example, timetabling and staffing constraints, and a lack of integration into the school curriculum [42]. Although RAW-PA was designed to be implemented outside of school and independent of the school curriculum, teachers identified that integrating elements of the program (eg, use of the Fitbit to track activity) within the existing HPE curriculum has the potential to provide leverage for organizational support and thus potentially increase sustainability. Institutionalization of physical activity interventions within the school system has previously been demonstrated for a health education intervention [43], although in-service teacher training was a key factor in the sustainability of such an intervention. Although RAW-PA teachers acknowledged the advantages of fewer implementation demands, teachers wanted greater involvement with and awareness of the program interactions with their students. Teachers also perceived RAW-PA to have positively influenced their own and the adolescents’ awareness of physical activity behaviors but recognized that community links may be required for broad program acceptability and thus sustainability in schools. If RAW-PA were to be integrated as part of the existing school curriculum, a degree of staff training may be required. This would also enable teachers to evaluate the advantages and/or disadvantages of increased knowledge/awareness of the intervention and involvement in overall implementation, on their existing time demands. Not only may this enhance ongoing implementation, but it may further embed physical activity promotion within school settings. Future research could explore the benefits of shared involvement between teachers, adolescents, and members of the school community in interventions such as RAW-PA on program effectiveness and long-term institutionalization in this context.

At an organizational level, factors known to enhance the implementation of physical activity interventions include the structure or policies within schools, resources available to support interventions, and the school climate [44]. For example, teachers questioned how such a program could be funded, given the costs associated with purchasing wearable activity trackers. Lower cost wearable activity trackers are currently available that may be purchased for use, either by students individually or as part of entry into a step challenge, for example, but this may still limit uptake into such programs. Teachers also flagged that there was a potential conflict between the RAW-PA delivery format and existing school policies restricting mobile phone use in schools. Adolescents also perceived the receipt of mobile phone–related program content (eg, email and/or text messaging alerts) during class time less favorably. Intermittent participant notifications about RAW-PA content were a central feature of the intervention, which were delivered predominantly outside of school hours to minimize any potential disruption during the school day. Nonetheless, where and when adolescents downloaded such notifications (eg, open Facebook to retrieve notifications) could not be controlled. As a result, sustainable implementation or system-wide institutionalization of RAW-PA may be substantially limited. Internationally, several countries and jurisdictions have implemented bans on mobile phones in schools, and such measures are increasingly being considered elsewhere [45-47]. Without modifications to the program design, such as changes to the volume and timing of program-related notifications, in its current format, RAW-PA may be a less feasible strategy for further implementation in schools or at scale.

### Strengths and Limitations

The strengths of this study are the larger sample size in comparison with previous wearable activity tracker interventions in adolescents, intervention duration, and collection of mixed methods data at multiple levels to understand implementation outcomes. The study was co-designed with the end users (adolescents); therefore, the potential translatability of the findings into practice at a larger scale was considered from the outset. However, the study is not without limitations. Although we considered sex differences during data collection, we were unable to consider sex differences in both the Facebook engagement data and focus group data due to restrictions with data identification. The Facebook engagement data were not split by sex; thus, we cannot comment on any differential use of Facebook or web-based resources by males and females. Restrictions on the Facebook data available for download also meant that it was not possible to determine if the same participant had viewed a Facebook post on more than one occasion. As such, the degree of engagement is based on the sum of all registered participants’ *views*, which may underestimate the level of individual engagement for some and overestimate it for others. Focus group data did not contain any identifying data; therefore, sex differences in the perspectives of the adolescents could not be examined further. Given that males and females engage differently with social media [14], it is recommended that future studies investigate any potential disparities and aim to capture data of this detail. The reported technical difficulties associated with the Fitbit Flex, app, and web-based platform may also mean that some adolescents who used their Fitbits were unable to, or chose not to, sync their data or access Facebook. As a result, this may have led to an underestimation of active adolescent engagement in the study. Second, 3 of the focus groups conducted included more than 10 participants, which may have impacted the extent to which each participant could put forth their views and experiences.

### Conclusions

RAW-PA showed good acceptability, but engagement and adherence were low among inactive adolescents living in socioeconomically disadvantaged areas. The intervention had high acceptability among teachers at the school level. There was evidence for self-reported perceived short-term positive effects on physical activity motivation and awareness, although these behavior changes were unlikely to be sustained. Low levels of teacher burden enhanced their perceptions concerning the feasibility of intervention delivery. However, sustainable implementation and institutionalization of digital behavior change interventions in schools may be limited by policies restricting the use of mobile phones in schools. Future research exploring the feasibility of differential strategies to engage young people with wearable technology interventions and ways of maximizing system-level embeddedness of interventions such as RAW-PA in practice would greatly advance the field.

## Abbreviations

HPE: health and physical education
MVPA: moderate- to vigorous-intensity physical activity
RAW-PA: Raising Awareness of Physical Activity
SEIFA: socioeconomic index for areas
